# Mapping genetic interactions in cancer: a road to rational combination therapies

**DOI:** 10.1186/s13073-019-0680-4

**Published:** 2019-10-22

**Authors:** Beril Tutuncuoglu, Nevan J. Krogan

**Affiliations:** 1 Department of Cellular and Molecular Pharmacology, University of California, San Francisco, 16th Street, Mission Bay Campus, San Francisco, CA 94158-2140 USA; 2 The J. David Gladstone Institutes, Owens Street, San Francisco, CA 94158 USA; 3 Quantitative Biosciences Institute, University of California, San Francisco, 4th Street, San Francisco, CA 94158 USA; 4 Cancer Cell Map Initiative (CCMI), La Jolla and San Francisco, CA USA

## Abstract

The discovery of synthetic lethal interactions between poly (ADP-ribose) polymerase (PARP) inhibitors and BRCA genes, which are involved in homologous recombination, led to the approval of PARP inhibition as a monotherapy for patients with *BRCA1/2*-mutated breast or ovarian cancer. Studies following the initial observation of synthetic lethality demonstrated that the reach of PARP inhibitors is well beyond just *BRCA1/2* mutants. Insights into the mechanisms of action of anticancer drugs are fundamental for the development of targeted monotherapies or rational combination treatments that will synergize to promote cancer cell death and overcome mechanisms of resistance. The development of targeted therapeutic agents is premised on mapping the physical and functional dependencies of mutated genes in cancer. An important part of this effort is the systematic screening of genetic interactions in a variety of cancer types. Until recently, genetic-interaction screens have relied either on the pairwise perturbations of two genes or on the perturbation of genes of interest combined with inhibition by commonly used anticancer drugs. Here, we summarize recent advances in mapping genetic interactions using targeted, genome-wide, and high-throughput genetic screens, and we discuss the therapeutic insights obtained through such screens. We further focus on factors that should be considered in order to develop a robust analysis pipeline. Finally, we discuss the integration of functional interaction data with orthogonal methods and suggest that such approaches will increase the reach of genetic-interaction screens for the development of rational combination therapies.

## Background

Whole genome and exome sequencing have provided an encyclopedia of genes that are involved in cancer development and progression, as part of programs such as The Cancer Genome Atlas (TCGA). These heroic efforts have revealed that many cancer cells hijack defined signature cancer pathways through acquired mutations that activate oncogenes or inactivate tumor suppressors [1]. Yet, these efforts have also demonstrated that the genetic backgrounds of different types of cancers are vastly heterogeneous, resulting in a high number of cases with inaccurate prognosis and ineffective chemotherapy treatments. Precision cancer therapeutics, which aims to tailor a treatment regimen to the unique genetic background of each disease, is a targeted and promising approach. This strategy relies on targeting particular mutants upon exploiting their genetic dependencies through the identification and mechanistic characterization of the genetic interactions involved in tumorigenesis, treatment response, and the development of drug resistance.

Genetic interaction occurs when pairwise perturbations of two genes involved in the same or parallel pathways result in a phenotype that is different from the expected additive effect of each individual mutation [2–4]. Genetic (epistatic) interactions can be synergistic (or synthetic), where the interaction of two genes exaggerates the phenotype, or buffering, where the perturbation of one gene masks the perturbation of another. Genes that result in a synergistic effect are commonly interpreted as working in compensatory pathways. The identification of such functional networks is particularly important for understanding oncogenic pathways because the heterogeneity in the genetic background of cancers is often associated with the connected pathways that might provide multiple potential rewiring mechanisms. Large-scale assessment of genetic interactions to identify functional networks has been performed using high-throughput assays in model organisms. One such example, in yeast, is the epistatic mini array profile (E-MAP) approach, which uses a symmetric matrix of gene perturbations to enable quantitative analysis of the type and strength of the interaction between each pair of genes that have been deemed to be functionally or physically related [5–8]. Hierarchical clustering analyses of the scores obtained from these genetic-interaction screens reveal functionally related genes and complexes.

In this article, we discuss recent targeted, genome-wide, and high-throughput screening studies that have employed dual loss-of-function, chemical–genetic interaction, and combinations of gene activation and inhibition methods to identify relevant genetic interactions. We also review the clustering and analysis pipelines used in high-throughput genetic-interaction screens for rapid translation of the generated data into effective therapies for cancer treatment. Furthermore, we suggest that combining genetic-interaction screens with orthogonal quantitative approaches to generate global networks can facilitate the development of rational combination therapies.

## Genetic interactions as therapeutic targets in cancer

Cancer cells often obtain selective advantage through functionally cooperative genetic interactions, in which the deleterious effects of oncogenic or tumor suppressor mutations are, presumably, compensated for by secondary alterations. For example, cancer cells can tolerate higher levels of replication stress that result from the overexpression of oncogenes because of the amplification of replication stress response kinases, such as ataxia telangiectasia mutated (ATM) and Rad3-related (ATR) kinase [9, 10]. Efforts by TCGA revealed such co-occurring and mutually exclusive genomic alterations in cancer. In this context, co-occurring mutations are potential candidates for dependency factors, while mutually exclusive alterations are potential candidates for synthetic lethality. Yet, it is important to emphasize the possible limitations of such approaches for functional interpretation. First, the differential classification of functional genetic variants to distinguish these from random passenger variants is not trivial. Second, sequencing results are not reflective of the protein levels or post-translational modifications in the cell. Even though the mutation of two genes may appear to be mutually exclusive at the genomic level, investigation of their final protein products may indicate a tendency for co-occurring alterations.

Inhibition of gain-of-function mutations in oncogenes is an effective cancer therapy approach, but restoring the functions of loss-of-function mutations in tumor suppressors is not yet clinically feasible. Rather than functional restoration, a strategic approach to exploit such mutations is to identify synthetic lethal interactions of tumor-suppressor genes in order to target tumor cells. Synthetic lethality is a form of synergistic genetic interaction, in which simultaneous deletion of two genes results in cell death whereas deficiency of either one of the same genes does not. Specific synthetic lethal interactions between the driver mutations of a tumor and druggable targets have been exploited to develop effective cancer treatments. For example, drugs targeting poly(ADP-ribose) polymerase (PARP) enzymes are synthetically lethal with loss-of-function mutations of *BRCA1* and *BRCA2* in tumor cells, leading to cell death resulting from the homologous recombination repair defects [2, 11–13]. PARP1 is a DNA damage sensor that binds to DNA damage sites, leading to the poly ADP-ribosylation (PARylation) of target proteins for the recruitment of DNA repair effectors. In addition, PARP1 auto-PARylation mediates its own release from the DNA damage sites [14]. PARP1 is also implicated in the reversal and repair of blocked replication forks [15]. Inactivation of the catalytic activity of PARP1 disrupts single-stranded DNA damage repair and causes PARP1 trapping by impairing its own release from the DNA damage site. These events block the replication fork reversal and cause double-stranded DNA breaks [15]. In cells that have a deficiency in homologous recombination repair, PARP1 trapping results in double-stranded lesions and eventually leads to cell death, providing an opportunity for targeted therapy in *BRCA*-mutant cancer cells (Table 1).

**Table 1:** Phase 3 or 4 clinical trials based on synthetic lethal and synergistic effects from genetic-interaction screen approaches^a^

Genetic interaction	ClinicalTrials.gov reference	Tumor	Results available
Synthetic lethality between PARP inhibition and BRCA1/BRCA2	NCT01945775	Breast	Yes
	NCT03150576	Breast	No
	NCT02163694	Breast	No
	NCT02184195	Pancreatic	No
	NCT01874353	Ovarian	Yes
	NCT02855944	Ovarian	No
	NCT01905592	Breast	No
	NCT02975934	Prostate	No
	NCT01844986	Ovarian	Yes
	NCT01847274	Ovarian	Yes
	NCT03863860	Ovarian	No
	NCT02000622	Breast	Yes
	NCT02502266	Ovarian	No
Synergy between BRAF inhibition and MEK inhibition^b^	NCT01584648	Melanoma	Yes
	NCT01682083	Melanoma	Yes
	NCT01245062	Melanoma	Yes
	NCT01597908	Melanoma	Yes
	NCT01909453	Melanoma	No
	NCT02967692	Melanoma	No
	NCT03551626	Melanoma	No
	NCT01689519	Melanoma	Yes
	NCT03273153	Melanoma	No
	NCT03340506	Melanoma, lung, glioma	No
Synergy between EGFR inhibition and BRAF inhibition^b^	NCT02928224	Colorectal	No

^a^Information accessed October 2019. ^b^Study conducted in a *BRAF*-mutant background. *EGFR epidermal growth factor receptor*

The use of PARP1 inhibitors as monotherapies for patients with *BRCA*-mutated cancer demonstrates how effective synthetic-lethality screens can be for drug development. Yet, as with many other therapies, resistance to PARP1 inhibitors arises in advanced disease, suggesting that the most effective responses to treatment with PARP1 inhibitors might be elicited either in early-stage disease or through the development of rational drug combinations [16]. To address both of these issues, several clinical trials are currently evaluating the efficacy of therapies that combine PARP1 inhibitors with chemotherapy or mutation-specific inhibitors (ClinicalTrials.gov reference NCT02576444) [17]. The PARP inhibitor niraparib was also tested for use as maintenance therapy in platinum-sensitive ovarian cancer, regardless of its *BRCA1* status [18]. The median duration of progression-free survival was significantly longer for patients receiving niraparib. These results, together with the observation that about 50% of epithelial ovarian cancer patients without *BRCA1* mutations exhibit defective homologous recombination, already indicate the potential wider reach of these PARP inhibitor therapies [19].

The dynamic rewiring of cancer cells that are exposed to anticancer drug treatments adds an additional layer of complexity to traditional functional interaction studies. In the clinic, the targeting of multiple factors within the same pathway has proven to be an effective strategy, possibly because targeting a signaling pathway can result in differential responses depending on the presence of upstream mutations [20, 21]. Moreover, therapy-resistance mechanisms in tumor cells rely on compensatory pathways that functionally buffer the inhibition of drug target genes. An example of this is the acquired resistance of *BRAF*V600E-mutant melanoma cells to BRAF inhibitors that occurs as a result of MAPK pathway activation. In this case, specifically in the *BRAF*V600E-mutant background, melanoma patients treated with a combination of a BRAF inhibitor with a MEK inhibitor exhibited improved progression-free survival when compared to patients treated with BRAF inhibitor alone [20–22] (Table 1). Combination therapy to target both the primary target and the resistance mechanism has been further supported as an effective strategy. A short hairpin RNA (shRNA) screen of human kinases and several kinase-related genes revealed that knockdown of *epidermal growth factor receptor* (EGFR) synergized with PLX4032, a BRAF inhibitor, in the suppression of *BRAF*V600E mutant colorectal cancers [23]. A phase 3 clinical trial recently demonstrated that a combination of encorafenib (a BRAF inhibitor), binimetinib (a MEK inhibitor), and cetuximab (an EGFR inhibitor) had an overall response rate (ORR) of 48% in *BRAF*V600E-mutant metastatic colorectal cancer patients, which was an increase in ORR compared to controls [24].

The development of high-throughput genetic-interaction screens with robust analysis and clustering pipelines is thus imperative to accelerate the identification of new druggable synthetic-lethal or other genetic interactions and to guide the improved prediction of drug synergies and rational combination drug therapies.

## Cancer models in mammalian cells and their applications in anticancer drug discovery

The key driver mutations causing oncogenesis and the factors involved in rewiring cancer cells in response to therapy remain unclear. Systematic and high-throughput approaches to dissect these functionally interconnected pathways might be clinically beneficial. Recent efforts to identify genetic interactions in a high-throughput platform involve combinatorial pairwise perturbations of two genes in an arrayed or genome-wide format (Table 2). The most common approaches to date are pairwise gene knockouts or a combination of a gene knockout and drug inhibition. A more recent and less-explored approach is to combine gene activation with gene inhibition, although the activation of a mutated gene is currently not feasible in the clinic.

**Table 2:** Comparison of different methods used to map genetic interactions

	Technique	Strength	Limitation	Considerations
Loss-of-function	shRNA, RNAi or CRISPRi	Allows investigation of essential genes Phenotype is reversible	Phenotype is gene-dosage dependent	Essential genes that are specific to a particular cell type are of interest
	CRISPR-Cas9	Allows investigation of complete functional shutdown	Ploidy in cancer cells may make the complete knockout of the gene difficult	Combinatorial or multiplexed knockouts enable investigation of the phenotypic effects of disrupting multiple genes at once
	Chemical inhibition	Allows direct investigation of therapeutic relevance	Dynamic range is dependent on drug dosage and treatment duration	Chemical-inhibition-based screens provide information on the mechanisms of action of the drugs
Gain-of-function	CRISPRa	Allows investigation of gain-of-function mutations	Feasibility beyond the K562 cell line is not clear	Combinations of CRISPRa and CRISPRi screens provide information on directionality of GIs
Screening approaches	Targeted or arrayed GI screening	Gene-editing efficiency can be analyzed by Sanger sequencing Enables straightforward exploration of multiple cell lines and conditions Amenable to the incorporation of more mechanistically informative phenotypes (e.g. using single-cell RNA-seq or imaging technologies)	Requires information on the genes and pathways of focus	Milder phenotypes may inform rational combinatory therapy designs
	Genome-wide GI screening	Allows determination of functional relations between previously unexplored gene pairs	Gene-editing efficiency is analyzed by next generation sequencing Requires increased computational bandwidth	Clustering analysis may enable identification of novel multi-molecular modules

*CRISPRa* CRISPR activation, *CRISPRi* CRISPR inhibition, *GI* genetic interaction, *RNAi* RNA interference, *shRNA* short hairpin RNA

### Dual loss-of-function methods

Dual loss-of-function studies form the foundation of genetic-interaction studies. Pairwise genetic-interaction screens in mammalian cells can involve the pairwise knockdown of specific genes using short interfering RNA (siRNA) or CRISPR inhibition (CRISPRi) platforms (where a catalytically dead version of Cas9 is fused to a Krüppel-associated box (KRAB) transcriptional repression domain) [25, 26]. Downregulation of target genes can result in a partial phenotype, so this approach can be used advantageously to target genes that are essential for viability [27]. Alternatively, combinatorial gene knockouts in mammalian cells can be mediated using the CRISPR-Cas9 platform [28, 29]. For example, Shen et al. [30] developed a systematic approach to map genetic networks by combining CRISPR-Cas9 perturbations. Pairwise gene knockout combinations of 73 cancer genes with dual-guide RNAs in three human cell lines—HeLa (human papilloma virus-induced cervical adenocarcinoma cells), A549 (an adenocarcinomic alveolar basal epithelial cell line), and HEK293T (human embryonic kidney cells)—enabled the identification of interactions that have potential therapeutic relevance. These interactions were then tested with drug combinations in order to develop synthetic-lethal therapies [30]. Interestingly, only 10.5% of identified interactions were common to given cell-line pairs, and no shared interactions were seen in all three cell lines. These observations might suggest a high degree of diversity in genetic interactions between different tumors, demonstrating the necessity of using a large number of cell lines and samples when performing similar studies.

Combinatorial CRISPRi screening platforms have been used to increase the throughput of approaches in which individual genes or gene pairs are downregulated [31, 32]. The proof of concept experiment, which targeted 107 chromatin-regulation factors in human cells using a pool of double-sgRNA constructs for the pairwise downregulation of genes, revealed both positive and negative genetic interactions [31]. In this context, it is important to confirm the repression efficiency of each combination of single-guide RNAs (sgRNAs) because the efficiency of double-sgRNAs was observed to be lower than that of single-sgRNAs [31]. This study was followed by a large-scale quantitative mapping of human genetic interactions using a CRISPR interference platform, in which 472 gene pairs were systematically perturbed in two related human hematopoietic cancer cell lines (K562 and Jurkat) [32]. Interestingly, even though this experimental pipeline captured 79.3% of the interactions listed in the STRING (Search Tool for the Retrieval of Interacting Genes/Proteins) database for the tested genes, the vast majority of the highly correlated gene pairs (315 of 390 genetic interactions (GI) with GI correlation > 0.6) were not captured by STRING annotation [33]. These results are indicative of either a lack of physical interactions between these functionally related gene pairs or unidentified protein–protein interactions. Systematic gene ontology annotation of the emergent gene clusters enabled the identification of gene clusters that might be functionally related in K562 and Jurkat cells, and suggested new factors that are involved in vital processes such as ER protein trafficking and DNA synthesis. The epistasis analysis used in this study revealed that the accumulation of an endogenous metabolite intermediate, isopentenyl pyrophosphate (IPP), causes replicative DNA damage and therefore increases the dependence of cells upon an intact DNA damage response pathway. This finding suggests a potential combination-treatment strategy, which both targets the pathway that promotes the accumulation of IPP and at the same time exploits the newly acquired dependence of the tumor cells upon the DNA damage response pathway. These experiments illustrate the potential of genetic-interaction maps in revealing combinations of druggable target genes that do not have a known physical association.

### Mapping chemical–genetic interactions

Quantitative chemical–genetic studies, in which inhibition by a compound is combined with a gene knockdown or knockout, are an alternative to pairwise gene perturbations [34, 35]. For example, investigation of the influence of the knockdown of 612 DNA-repair and cancer-relevant genes on the response to 31 chemotherapy compounds revealed that loss-of-function mutations in *ARID1A* and *GPBP1* contribute to PARP inhibitor and platinum resistance in MCF10A, a non-tumorigenic human breast epithelial cell line [34]. This result is in contrast to the findings of another chemical–genetic screen that tested isogenic ARID1A-deficient MCF10A cells against a panel of chemotherapeutic drugs and DNA-repair inhibitors [36]. This screen indicated an increased sensitivity of ARID1A-deficient cells to a combination of ionizing radiation with PARP inhibition [36]. Inactivating mutations in *ARID1A* have been detected in multiple forms of human cancers. ARID1A is a component of the SWI/SNF chromatin remodeling complex and is implicated in non-homologous end joining (NHEJ), suggesting that it might be an important modulator of the response to PARP inhibitors and combination therapies.

Deep investigation of the genetic targets of therapies that have already been approved by the US Food and Drug Administration has the potential to expand the number of patients who can benefit from these therapies by revealing novel targets that are highly mutated in cancer cells. For example, further investigation of the synthetic lethality of PARP inhibitors with *BRCA*1 and *BRCA*2 mutations instigated a series of discoveries that suggest that PARP inhibitors can also be used to target deficiencies in other genes that are involved in homologous recombination [37–40]. Several studies investigated the synthetic lethal interactions of PARP inhibitors [11, 41] and ATR inhibitors [9, 42] against custom siRNA libraries. The clinical relevance of these studies is currently being tested in clinical trials of multiple rational drug combination therapies (Table 1, ClinicalTrials.gov reference NCT04065269) [17, 43, 44]. In addition to defects in genes involved in homologous recombination, mutations in other genes have also been shown to sensitize cancer cells or immortalized cells to PARP inhibitors. Recently, a genome-wide dropout CRISPR screen for genes that, when mutated, sensitize cells to PARP inhibition was performed using the human cell lines HeLa, RPE1-hTERT (a telomerase-immortalized retinal pigment epithelium cell line), and SUM149PT (a triple-negative breast cancer cell line with *BRCA*1 mutation). Dropout screens are generally used to identify genes that are essential for cell viability, and they involve RNA interference (RNAi) or CRISPR screening of two or more cell lines over a series of cell divisions. In this case, the screen revealed the hypersensitivity of RNase-H2-deficient cells to PARP inhibition [35]. Of 155 high-confidence gene knockouts that sensitized cells to the PARP inhibitor olaparib, 13 genes scored positive in all three cell lines, and 60 genes were common to two cell lines. Besides the factors that are known to be involved in homologous recombination and Fanconi anemia, and the kinases ATM and ATR (which are involved in the DNA damage response), genes encoding splicing and transcription factors and the RNase H2 enzyme complex were shown to sensitize cells to olaparib treatment in all three cell lines. A parallel screen utilized a similar genome-wide CRISPR-Cas9-based approach in three independent human cell lines to identify genes that, when depleted, showed synthetic lethality with ATR inhibition [45]. Interestingly, depletion of the RNAse H2 enzyme also led to a synthetic lethality with ATR inhibition. Collectively, these data indicate that loss of RNase H2 might be a promising biomarker for PARP and ATR inhibitor-based therapy, and provide an opportunity for a rational combination therapy involving PARP and ATR inhibitors for RNase H2 loss.

An orthogonal strategy, which has the simultaneous advantage of increasing the throughput of screens, is to leverage the conserved interactions in model organisms. Large-scale genetic-interaction screens have been developed in the yeasts *Saccharomyces cerevisiae* and *Schizosaccharomyces pombe*, and have been used extensively to gather biological insights [5, 46–48]. However, the genetic interactions observed in model organisms need to be validated in mammalian cells and in the clinic. Thus, a viable hybrid approach is to target conserved tumor suppressor genes for genetic interactions in yeast, followed by validation of the identified interactions in mammalian cells. For this purpose, synthetic genetic array (SGA) analysis provides a convenient and large-scale platform for systematic construction of double mutants in yeast, allowing the mapping of synthetic genetic interactions. SGA involves the construction of double mutants by crossing a query mutant strain to an array of approximately 5000 viable deletion mutants [48]. In order to connect tumor suppressor genes to druggable targets, Srivas et al. [49] used SGA technology in *S. cerevisiae* and constructed a genetic-interaction map of 43,505 gene pairs that are known to be small molecule targets, tumor suppressors, or clinically relevant [49]. Guided by the yeast network, a more targeted chemo-genetic interaction map obtained using 21 drugs and 112 tumor suppressor genes in HeLa cells revealed a total of 127 synthetic sick or synthetic lethal interactions. Clonogenic assays were then performed to determine whether the interactions identified in the chemo-genetic screen (on the basis of an observed reduction in cell growth) also resulted in the reduced survival of individual tumor cell clones. Five of the seven combinations identified from the conserved tumor suppressor XRCC3 network resulted in negative effects on tumor cell clonal survival when *XRCC3* is also knocked down. XRCC3 is involved in the homologous recombination repair pathway. These results suggest that the drugs targeting relevant genes should be investigated as therapies for tumors with *XRCC3* loss-of-function mutations.

### Mapping the directionality of genetic interactions

Functional and modular data obtained through genetic-interaction methods can fall short in providing information about directional and regulatory dependencies. Orthogonal approaches that can be incorporated with the genetic-interaction data to overcome this limitation are discussed in the next sections. This shortcoming has been addressed by several studies. For example, in combinatorial RNAi screens conducted in *Drosophila* cells, regulatory and temporal directionality was derived through mathematical modeling and time-dependent analysis of differential genetic interactions [50, 51].

A recent quantitative dual screen addressed this issue by combining the CRISPR-mediated activation (CRISPRa) of one gene with the knockout of a second gene [52]. This combinatorial approach has the additional advantage of enabling studies of the effects of gene amplification or gain-of-function alterations of several proto-oncogenes, which are known to be just as important as the effects of gene deletions for rewiring of cancer cells. This enabled the formation of a directional dependency network for human K562 leukemia cells. The systematic identification of genes whose activation altered the fitness of K562 cells treated with the tyrosine kinase inhibitor imatinib was conducted using a genome-wide library targeting every coding and over 4000 non-coding transcripts [52]. In addition to genes with known roles in leukemia and imatinib resistance, this screen identified previously uncharacterized genes (*BBX*, *NOL4L*, and *ZC3HAV1*), which were shown to have roles in drug resistance. To quantify dual genetic interactions, activating sgRNAs targeting 87 candidate genes from the primary screen were combined with knockout sgRNAs targeting 1327 genes from KEGG-annotated cancer-relevant signaling pathway genes. The directional dependencies of the genetic interactions were then inferred for those cases in which one gene activated its partner. In these gene pairs, individual activation and knockout of the activating gene partner produce opposing phenotypes, providing an opportunity to incorporate this information into the genetic-interaction scoring algorithm that accounted for the singular and combinatorial perturbation phenotypes. Such a high-throughput approach enables the identification of genes that can be exploited for cancer therapy. As this approach has been limited to K562 cells, it still remains to be explored whether this method is widely applicable to other models.

## Considerations for a robust analysis pipeline

The inference of functional data from large-scale genetic network mapping in human cells requires robust and thorough data-analysis pipelines. In this context, a data-analysis workflow involves considerations for experimental design, quality control, and mathematical scoring. The earliest studies on the use of genetic-interaction mapping to dissect the functions of protein complexes involved E-MAPs in yeast [47, 53, 54], as mentioned earlier. These studies established the ground rules in terms of experimental design for isolating hits and building a reliable genetic-interaction map. The computational scoring and clustering algorithms used to analyze the data include statistical analyses of the strength of each genetic interaction, of the correlation between replicates, and of the clustering of biological complexes [55]. Similar computational scoring algorithms can be applied to mammalian systems.

In mammalian systems, several high-throughput genetic-interaction screens have been conducted using a targeted approach with some prior knowledge on the interaction networks of the genes or the pathways to be studied [30–32, 34, 49]. This kind of approach decreases the noise and minimizes the potential for false negatives in the data, allowing milder phenotypes to be detected. Even though these milder phenotypes might not be good targets for monotherapy, they might provide clues for combinatorial drug design and about functional redundancy in cancer cells. A promising strategy for combinatorial drug discovery is to target compensatory pathways to block functional redundancies. With the current methodologies, genome-wide trigenic interaction mapping is not trivial, but these milder phenotypes can be used to predict target candidates for combinatorial drugs and can be tested in combinatorial, trigenic contexts [56]. As compared to targeted screens, genome-wide analysis allows the unbiased determination of genetic interactions without prior knowledge of physical or functional networks [45, 57, 58]. Genome-wide screens have a potential to reveal unexpected interactions between previously uncharacterized gene pairs (Table 2).

However, any CRISPR-Cas9 based genetic-interaction analysis comes with three major considerations. First, there is heterogeneity in the editing efficiencies provided by different sgRNAs. This consideration applies to CRISPR-Cas9-based screens performed either in an arrayed format or as pooled libraries. In addition to using at least three sgRNAs for each targeted gene, quantitative assessment of gene-editing efficiency in arrayed knockout experiments should be conducted using tools such as TIDE (Tracking of Indels by Decomposition) or using ICE (Inference of CRISPR Edits) analysis following Sanger sequencing [59–61]. Once the gene-editing efficiency for each sgRNA is confirmed, the genotype–phenotype correlation in arrayed formats is straight forward. In comparison, the analysis of genome-wide pooled screens requires the use of next-generation-sequencing (NGS) technologies for genotype–phenotype correlation.

The second consideration is cell-line variability. The Cancer Genome Atlas (TCGA) dataset indicates that 89% of tumors, of 33 cancer types, contain at least one somatic driver alteration in ten canonical signaling pathways that are known to be highly mutated in cancer [1]. These data represent commonalities between different cancer types. Yet, predictions of disease prognosis and drug sensitivity in cancer are vastly inaccurate because of the diverse mutational landscape of individual tumors. For example, a recent study suggested that the tumor lineage determines whether mutations in *BRCA*1 and *BRCA*2 are indispensable founding events or biologically neutral events for tumorigenesis [62]. In addition, the genomic copy number of different cell lines was suggested to affect CRISPR targeting and toxicity after genome editing [63, 64]. These observations are indicative of the importance of conducting functional interaction screens in multiple different cell lines, not only for the identification of robust synthetic lethal or other interactions, but also for the identification of more targeted precision treatment opportunities.

Third, drug dosing and timing should be considered. Importantly, for screens that measure phenotype upon drug treatment, the dynamic range of experiments is highly dependent on the drug concentration and treatment duration. Boettcher et al. [52] showed that, when compared with a single treatment, repeated drug treatment can allow for greater enrichment of resistance genes. For chemogenetic interaction profiling that accounts for the stated considerations, drugZ scoring has been introduced as a software tool for the identification of both synergistic and suppressor interactions [35, 65].

## Combining genetic interaction screens with orthogonal quantitative approaches to generate global networks

**Fig. 1:**
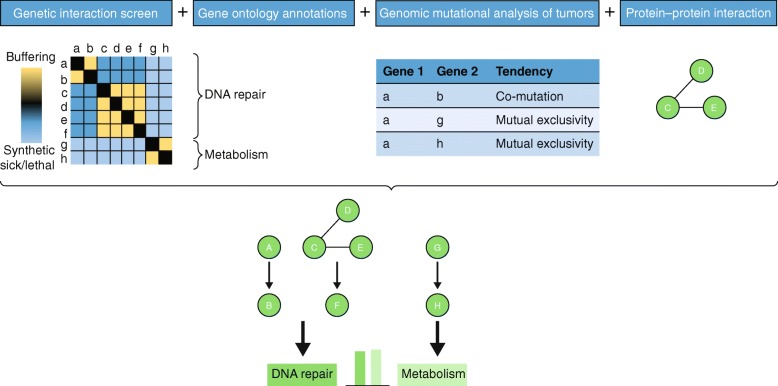
Hypothetical integration of genetic-interaction screens with orthogonal quantitative approaches to enable the identification of pathways. From *left* to *right*, the experimental pipeline is such that genetic interactions are scored and clustered to identify genes that are potentially involved in the same or parallel functionally relevant pathways and/or in potential protein complexes. These genes are annotated using Gene Ontology terms [66]. The mutational landscapes of the genes of interest are tested for statistically significant co-mutation or mutual exclusivity. A co-immunoprecipitation experiment is conducted to identify the proteins that interact with the protein encoded by the gene of interest. Data obtained through these orthogonal approaches are combined to deduce the biological pathway

A major goal of functional-interaction mapping studies is to elevate gene-association studies from the identification of individual genes that are associated with phenotypes to providing more interpretable genetic information on the biological pathways that are involved. In addition, the ability to combine functional interactions with physical interaction modules, in order to build global interaction networks, is important for dissecting the effects of differential mutations in cancer. High-throughput genetic-interaction screens generate an unprecedented amount of cell-specific functional genomics data that can help to reveal genetic networks. Genetic-interaction profiles provide a quantitative measurement of functional similarity. These maps can be overlapped with different kinds of network information obtained by orthogonal approaches to further inform functional interpretation and the prediction of novel gene function (Fig. 1) [67]. These approaches include gene-ontology analysis, as well as analyses of the mutational landscape of patient tumors, gene regulation, and protein–protein interaction.

Gene-ontology analysis provides a platform for the systematic annotation of the gene clusters that are enriched for genes known to act in similar pathways or in a given complex [32, 68]. Statistical analysis of the genomic mutational landscape of patient tumors from TCGA provides an additional layer of information, as gene pairs that are rarely co-mutated are candidates for synthetic lethal interactions [69–71]. In addition, because cancer cells are under selective pressure, two genes might need to be co-mutated to provide a growth advantage to tumor cells. Yet, as discussed earlier, these approaches for functional interpretation are statistically limited by the small number of tumors that have been sequenced and by the unclear classification of functionally relevant mutations. Integrating co-expression data and gene-regulation information from gene expression profiles can also be a useful approach for establishing correlations and extracting functional subnetworks. In particular, recent advances in the analysis of single-cell RNA sequencing data provide a reliable platform for the interrogation of gene–gene relationships [72–74]. Perturb-seq combines single-cell RNA-seq with pooled CRISPR-based gene perturbations, and this tool has been developed to obtain a greater amount of mechanistic information from genetic perturbation screens by the identification of gene targets through changes in gene expression [74]. Norman et al. [73] also applied this technology to the CRISPRa platform, and were able to determine the differential expression profiles of 112 genes whose activation resulted in growth enhancement or retardation in K562 human leukemia cells [73]. Finally, the incorporation of annotated protein–protein interaction data into genetic-interaction screens can enable the mapping of comprehensive global networks that include information at both the genomic and the proteomic level in the cell. Protein–protein interaction studies using multiple different cell lines can provide a network-level framework for differential genetic interactions that are observed in various cell lines [75].

Several recent studies have employed integrated network analysis to investigate the long-standing question of the involvement of virus infections in the development of cancer. Large-scale protein–protein and genomic screens addressed the roles of human papillomavirus (HPV) in oncogenesis and human lymphotropic virus type I (HTLV-I) in adult T cell leukemia/lymphoma (ATLL) [76, 77]. The physical interactions of HPV and human proteins in three different cell lines (C33A, HEK293, and Het-1A) were determined by mass spectrometry following the affinity purification of complexes associated with viral proteins. The protein–protein interaction data were then combined with data defining the genomic mutational landscape of tumors. Comparison of HPV^+^ and HPV^−^ tumor samples led to the identification of eight genes that are altered frequently in HPV^−^ tumors but rarely in HPV^+^ tumors. This finding was followed by the generation of a network propagation framework, in which proteins were scored on the basis of their proximity to HPV-interacting proteins or proteins that are preferentially mutated in HPV^−^ tumors within the Reactome functional interaction (ReactomeFI) reference network. This integrative approach resulted in the identification of an interaction between L2 HPV protein and the RNF20/40 histone ubiquitination complex that promotes tumor cell invasion [76, 78]. Around the same time, a pooled shRNA screen targeting lymphoid regulatory factors in eight ATLL cell lines revealed essential roles for the BATF3–IRF4 transcriptional network in malignant ATLL cell proliferation [77]. The gene expression profiles of BATF3 or IRF4 knockdowns overlapped significantly with each other, with 494 genes decreasing significantly. In addition, inactivation of HBZ, the HTLV-1 viral protein whose expression is maintained in all ATLL cells, resulted in decreased abundance of BATF3 and MYC mRNAs*.* ChIP-seq analysis revealed that MYC is a direct target of BATF3–IRF4, but not of HBZ, suggesting that HBZ regulates MYC expression through BATF3. Finally, the relevance of this type of analysis to the development of new treatments was tested by evaluating the sensitivity of ATLL cells to bromodomain and extra-terminal motif (BET) inhibitor JQ1. BET family proteins can regulate the expression of several oncogenes upon recognizing histone lysine acetylation to assemble transcriptional activators and chromatin-interacting complexes [79]. JQ1 treatment was toxic to the ATLL cells and reduced BATF3 and MYC mRNA levels in the cell. Currently, BET inhibitors are being studied extensively in clinical trials, both as monotherapy and in combination therapy to halt the transcription of oncogenes and to decrease cancer cell survival in multiple different cancer types [80].

## Conclusions and future directions

**Fig. 2:**
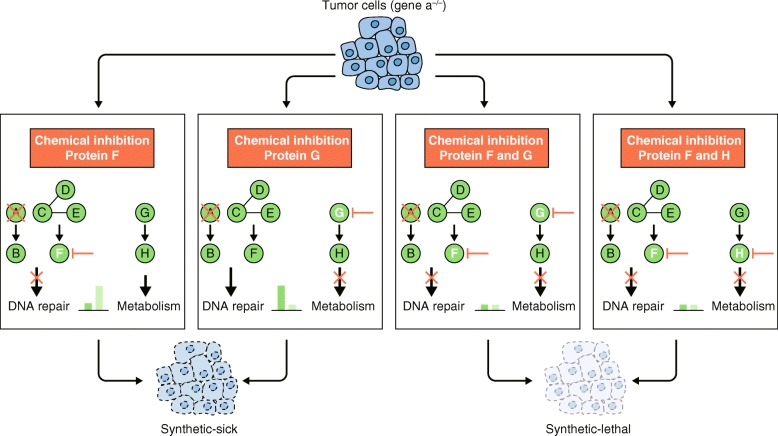
Strategy for a rational combination-therapy design. The interactions are based on the pathway from Fig. 1. A loss-of-function mutation in gene a is indicated to be a driver mutation for cancer development. The hypothetical case indicates a synthetic-sick interaction between gene a (which is involved in DNA repair) and gene g (which is involved in cellular metabolism). From *left* to *right*, inhibition of gene f or gene g in the cancer (a^−/−^) background results in synthetic sickness, but not lethality. Synthetic lethality in the cancer background is only achieved by co-inhibition of the genes f and g (or of genes f and h)

Genetic-interaction screens conducted in mammalian cells within the past couple of years have proven to be powerful approaches for the functional characterization of genes by determining novel genetic dependencies of genes or pathways, through dual loss-of-function or chemicogenetic analysis, respectively. The combination of CRISPR-based screening technologies and integrative analysis pipelines has enabled the formation of interaction networks that provide new insights into the functions of genes. Moreover, synthetic lethal or synthetic sick interaction pairs guide the design of selective combination therapies (Fig. 2). For example, mutations in several homologous recombination factors or inhibitors of the phosphatidylinositol 3-kinase signaling pathway, which were shown to synergize with PARP inhibition in BRCA1-proficient cancer cells in preclinical studies, are currently being tested in clinical trials (ClinicalTrials.gov reference NCT03344965). In line with this, buffering genetic interactions of drug target genes are candidates for drug-resistance mechanisms. Thus, the inhibition of these resistance mechanisms together with the primary genes may be an effective therapeutic strategy. It is imperative that genetic-interaction screens are expanded to include more genes and cell types to enable the identification of global networks. Comparisons of different cell types can reveal differences among cell types that can have important distinguishing biological implications.

To gain insights into the dynamic functional relationships between cellular processes and the rewiring of cancer cells in response to changing conditions such as drug treatment, it is important to consider differential genetic-interaction approaches in response to a stimulus. Most genetic-interaction analysis in mammalian systems is limited by ‘end-point’ experiments and by the use of non-specific phenotypic readouts, such as cellular growth rate. The analysis of genetic network plasticity and context-dependent rewiring events has been demonstrated in yeast and *Drosophila* cells, where quantitative comparisons of genetic interactions in untreated and treated conditions at different timepoints have revealed an enrichment of interactions in the target pathway [51, 81]. Similar dynamic rewiring events can also be revealed by time-resolved analysis following loss-of-function mutations in mammalian systems. Coupling CRISPR-based gene perturbations to more mechanistic readouts, such as proteomic, transcriptomic or cell-localization phenotypes, will also enable the mechanistic elucidation of epistatic interactions. A derivative approach that is yet to be conducted in high-throughput systems is the inference of drug-resistance mechanisms. These approaches would inform rational drug combinations and accelerate the development of targeted therapies.

To date, genetic-interaction screens in mammalian cells have relied on differential gene copy number and expression profiles in cancer cells and cell-proliferation readouts. Yet, most tumors arise as a result of a mutation rather than the complete absence of a gene [71]. Distinguishing driver mutations and their specific functions will facilitate the discovery of target pathways. Therefore, conducting gene-interaction screens using pathogenic mutant versions of the target genes, rather than complete gene knockouts, will be important for drug development.

Analyses of the mutational landscapes of tumors indicate that each tumor harbors a high number of somatic mutations. Global network analysis might reveal that these mutations converge in several hub events, such as protein interactions or transcriptional regulation. The integration of genetic-interaction datasets with other sources of information obtained through orthogonal experimental and computational tools is challenging and requires effective collaborations between molecular and cancer biologists, computational biologists, and clinicians. Several groups have formed such collaborative mapping initiatives in mammalian systems [73, 75, 82]. Ultimately, these efforts promise to lead to global network maps, which could allow predictions of effective drug–target combinations for each individual cancer cell background.

## Abbreviations

ATLL: Adult T cell leukemia/lymphoma
ATM: Ataxia-telangiectasia mutated kinase
ATR: ATM- and Rad3-related kinase
CRISPRa: CRISPR activation
CRISPRi: CRISPR inhibition
EGFR: *Epidermal growth factor receptor*
E-MAP: Epistatic mini array profile
GI: Genetic interaction
HPV: Human papillomavirus
HTLV-I: Human lymphotropic virus type I
IPP: Isopentenyl pyrophosphate
PARP: Poly(ADP-ribose) polymerase
PARylation: Poly ADP-ribosylation
RNAi: RNA interference
SGA: Synthetic genetic array
sgRNA: Single-guide RNA
shRNA: Short hairpin RNA
siRNA: Short interfering RNA
STRING: Search Tool for the Retrieval of Interacting Genes/Proteins
TCGA: The Cancer Genome Atlas
